# *Coxiella burnetii* infection in humans: to what extent do cattle in infected areas free from small ruminants play a role?

**DOI:** 10.1017/S0950268820001880

**Published:** 2020-08-26

**Authors:** M. Pouquet, N. Bareille, R. Guatteo, L. Moret, F. Beaudeau

**Affiliations:** 1 INRAE, Oniris, BIOEPAR, 44300, Nantes, France; 2 UMR 1246 INSERM SPHERE ‘MethodS in Patients-centered outcomes and HEalth ResEarch’, Universities of Nantes and Tours, Nantes, France; 3 Department of Public Health, Nantes University Hospital, Nantes, France

**Keywords:** Cattle, *Coxiella burnetii*, human, occupation-related infections, seroprevalence

## Abstract

Domestic ruminants (cattle, goats and sheep) are considered to be the main reservoirs for human *Coxiella burnetii* infection. However, there is still a need to assess the specific contribution of cattle. Indeed, most seroprevalence studies in humans were carried out in areas comprising both cattle and small ruminants, the latter being systematically implicated in human Q fever outbreaks. Therefore, we conducted a cross-sectional study in areas where *C. burnetii* infection in cattle was endemic, where the density of cattle and small ruminant farms were respectively high and very low. The aim was to estimate the seroprevalence rates among two occupational (cattle farmers and livestock veterinarians), and one non-occupational (general adult population) risk groups. Sera were collected in 176 cattle farmers, 45 veterinarians and 347 blood donors, and tested for phase I and II antibodies using immunofluorescence assay. Seroprevalence rates were 56.3% among cattle farmers, 88.9% among veterinarians and 12.7% among blood donors. This suggests that a specific risk for acquiring *C. burnetii* infection from cattle in endemically infected areas exists, mainly for occupational risk groups, but also for the general population. Further research is needed to identify risk factors for *C. burnetii* infection in humans in such areas.

## Introduction

*Coxiella burnetii* is a Gram-negative bacterium responsible for Q fever, a worldwide distributed zoonotic disease. The main animal reservoirs for human infections are domestic ruminants (cattle, sheep and goats). Q fever is as an occupational disease for people working in direct contact with potentially infected animals (e.g. livestock farmers, veterinarians and abattoir workers). However, the airborne dispersion of the bacteria also gives a potential for widespread contamination of the general population. *C. burnetii* is most commonly transmitted to humans through inhalation of contaminated aerosols issued from abortion and parturition products, faeces and urine of infected animals and their environment [1].

The large 2007–2010 epidemic in the Netherlands was proved to be linked to the exposure of humans to contaminated particles issued from infected dairy goat farms, experiencing abortion waves, and located in the surroundings; a large majority of cases involved the general population, while only 5% of Q fever patients had reported an activity related to agriculture [2]. In France also, the Q fever cases reported in the 2000s occurred mainly in regions with a high density of small ruminants and all the epidemiological investigations in farms in case of human outbreaks concluded about the implication of infected small ruminants [3].

By contrast, no associations between proximity or contact with infected cattle and Q fever cases in humans have been described during the period 1982–2010 in Bulgaria, France, Germany and the Netherlands [4]. Nevertheless, *C. burnetii* infection was reported to be widespread among Dutch dairy cattle herds (78.6%; [5]), as well as among humans living or working on dairy cattle farms (72.1%; [6]). However, the incidence rate of clinical cases among cattle farmers remained very low (0.5%) [6]. More generally, even in areas or countries where *C. burnetii* infection has been reported to be endemic in cattle and in humans who were occupationally exposed to cattle, the clinical cases remained rare [7].

A reason for the high level of seroprevalence in cattle farmers reported during the Dutch epidemic was that some farmers had been likely affected by the infected small-ruminant farms located nearby [6]. Thus, under the assumption that the seroprevalence rate among cattle farmers indirectly measures the implication of cattle in the spillover of *C. burnetii* to humans, the specific contribution of these animals might have been highly overestimated at least in the Dutch context. Indeed, a fraction of the farmers had a serological status, when positive, resulting from goat-related determinants, and being not attributable to their cattle management practices. The hypothesis of an overestimated role of cattle was supported by [8] who reported a much lower seroprevalence rate (3%) among 163 cattle farmers in Denmark, where only very few sheep and goat farms exist. To thoroughly assess the contribution of cattle, it is crucial to control this misclassification bias in the outcome of interest, due to the concomitant presence of cattle and small-ruminant farms within a same geographical area.

Therefore, we carried out a cross-sectional study purposively in areas where *C. burnetii* infection in cattle was endemic (to ensure putative spillover to humans nearby), where the density of cattle and small ruminant farms were respectively high and very low (to specifically assess the role of cattle), and where the annual incidence of notified clinical cases of Q fever was also very low (to assess the risk in the absence of previous outbreak), with the aim to estimate the seroprevalence rate of antibody-carriers against *C. burnetii* among occupational (cattle farmers and livestock veterinarians), and non-occupational (general adult population) at-risk groups. The two occupational populations were chosen in order to assess a possible exposure dose–response effect, by assuming that the durations and types of contacts with cattle differed between these two groups, the farmers being mainly exposed to one main source of infection (i.e. their herd), while the livestock veterinarians were *per se* exposed to multiple sources of infection and at-risk situations (such as abortions).

## Materials and methods

This study was approved by the Ethics Committee of Ambroise Paré Hospital (Comité de Protection des Personnes Ile de France VIII, Boulogne-Billancourt, France) and conducted in accordance with the Helsinki Declaration of 1975, revised in 2013, and with the French law for biomedical research (No. ID EudraCT/ID RCB: 2017-A01304-49).

### Study areas

**Fig. 1.**
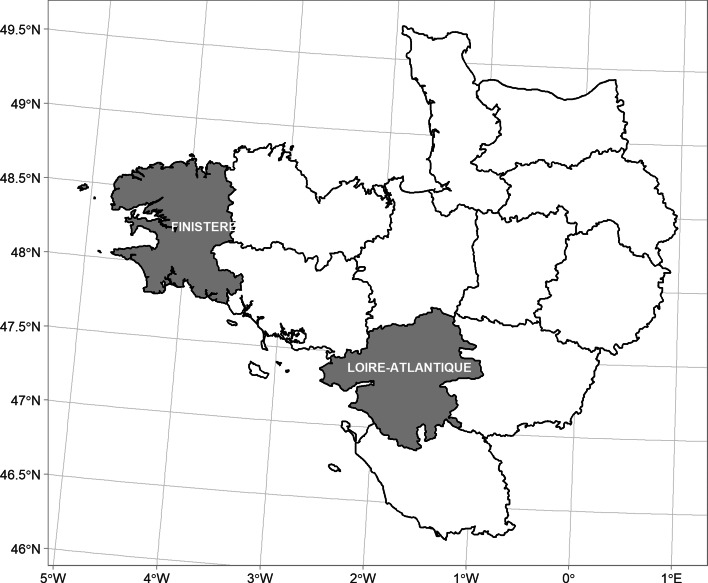
Location of the departments Finistère and Loire-Atlantique in western France.

The cross-sectional study was conducted from November 2017 to June 2018 in two departments (Finistère and Loire-Atlantique) located in western France (Fig. 1). These two departments met the following requested criteria to achieve the objectives: (1) a high density of cattle herds and low density of small ruminant herds; (2) no previous report of Q fever outbreaks and a low number of reported cases of human Q fever and (3) *C. burnetii* infection being highly endemic in cattle herds. In both departments, the density of cattle and of small ruminants were respectively high and low (>50 cows/km^2^ and <3 goats and ewes/km^2^). *C. burnetii* infection was endemic in cattle: the prevalence of dairy herds with seropositive animals was 69.4% in Finistère [9] and 56.1% in Loire-Atlantique respectively (unpublished data). In addition, the annual incidence of acute Q fever cases in humans was less than 1.5 cases/1 million inhabitants/year in both departments (overall mean in France: 4.5; maximum: 19) [3].

### Study populations and samples

The sample issued from the general adult population consisted of blood donors from Finistère. Participants were recruited during blood donations sessions organised by the EFS (Etablissement Français du Sang). The EFS regularly organises blood collection in fixed and mobile centres all over the Finistère department that allowed the recruitment of healthy subjects with urban and/or rural living experience. For the present study, the selection of collection centres on a given date (‘location-date’) was made (i) on the basis of operational feasibility criteria, according to the schedule provided by the EFS, (ii) so that their location ensures a large geographical coverage of Finistère and (iii) according to the population density of the canton in which the collection took place. At each ‘location-date’, voluntary blood donors were offered the opportunity to participate according to age and gender quotas, which were previously defined from the characteristics of the entire population of Finistère. Thus, the resulting sample was proportional to the distribution of age, gender and place of residence (rural or urban) of the general adult population. Assuming an expected minimum prevalence of 5% of seropositive subjects in a total population of 30 000 donors in Finistère, with an error of 2.5%, the minimum number of subjects needed was 290.

Cattle farmers were recruited with the support of the GDS 44 (Groupement de Défense Sanitaire – Animal Health Service – of Loire Atlantique) on a voluntary basis. All the dairy and beef cattle farms located in Loire-Atlantique, which housed at least 10 adult cows in September 2017, and were registered in the GDS44 database (*n* = 2224), were eligible and then invited by postal mail to participate in the study. Volunteers contacted the project team by email or by phone to confirm their participation. Assuming an expected minimum prevalence of 40% of seropositive farmers, with an error of 8%, the minimum number of subjects needed was 135.

All the livestock practitioners working in Loire-Atlantique and in Finistère (220 veterinarians identified from the national registration database) received by e-mail an invitation to participate in the study. Because of a low response rate at first contact, two reminders were made 1 month apart. Assuming an expected minimum prevalence of 50% of seropositive veterinarians, with an error of 10%, the minimum number of subjects needed was 50.

Whatever the populations, all participants had to fulfil the following inclusion criteria: acceptance to participate, being aged more than 18 years old, with no clinical symptoms suggestive of Q fever at the time of inclusion (both criteria being regulatory requirements to be blood donor) and with no prior history of Q fever vaccination (in the absence of any DIVA assays suited to differentiate vaccinated from naturally infected persons).

### Blood collection, serum sample preparation and analysis

Cattle farmers and livestock veterinarians who agreed to participate in the study were contacted by email and/or by phone to schedule a visit at their working place for collection. First, each participant who agreed and fulfilled the sampling criteria signed informed consent to participate. With regards to blood donors, an additional tube of blood was obtained at the time of collection by the EFS nursing staff. For farmers and veterinarians, blood was collected specifically by a private nurse.

Collected blood samples were transported in a biosafety container to the Centre Clinique d'investigation at Brest hospital (INSERM CIC 1412, CHRU Brest, France) for those taken in Finistère, and to the Centre de Ressources Biologiques at Nantes hospital (BB-0033-00040, CHU Nantes, France), for those taken in Loire-Atlantique, to be centrifugated (2200 ***g***, 15 min, 18 °C) within 5–6 h of collection. Sera were then frozen and transported at −80 °C to Eurofins Biomnis Clinical Trials Business unit (Lyon, France) for serological analysis. Serological analyses were performed using an immunofluorescence assay (IFA) (Focus Diagnostics, Cypress, CA, USA), to test serum samples for *C. burnetii* phase I and II IgG, following the manufacturer's instructions. The screening dilution was of 1:16. Participants with a positive phase II IgG result were classified as seropositive, to allow the detection of current or past infections, as IgG appears almost simultaneously with IgM in the case of *C. burnetii* infection and can persist over years [10]. Participants with all other outcomes were classified as seronegative.

For each population, the seroprevalence rate was calculated as the number of subjects classified as seropositive over the total number of subjects in this group.

## Results

A total of 568 participants were included in the study: 347 blood donors, 176 cattle farmers and 45 livestock veterinarians. The three groups did not statistically differ according to age (mean (s.d.): 49 (15), 50 (10) and 45 (13) years-old among blood donors, cattle farmers and veterinarians, respectively). There were much more men in occupational groups compared to blood donors (48% among blood donors, 81 and 75% among cattle farmers and livestock veterinarians respectively).

**Fig. 2.**
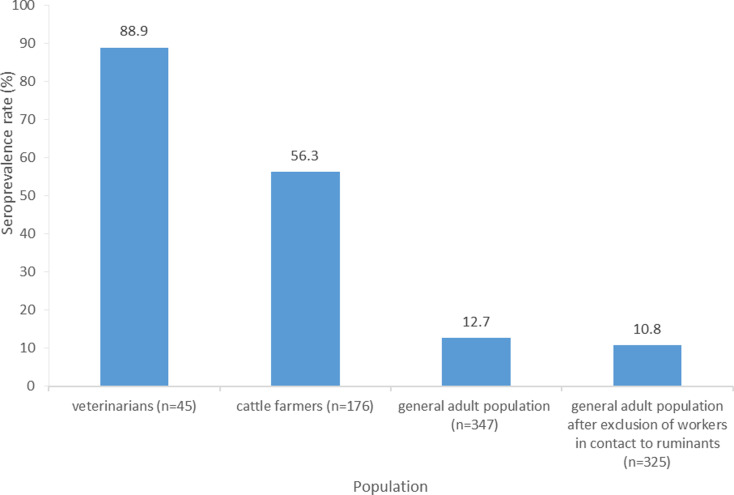
Seroprevalence rates of antibody-carriers against *C. burnetii* among livestock veterinarians, cattle farmers and the general adult population.

The number of seropositive subjects in each group was as following: 44 blood donors, 99 farmers and 40 veterinarians. Among the 22 blood donors who worked in contact with ruminants, nine were seropositive. The corresponding seroprevalence rates are displayed in Figure 2. The estimated seroprevalence rates were higher among livestock veterinarians (88.9%; 95% confidence interval (CI): [79.7–98.1]) and cattle farmers (56.3%; 95% CI: [48.9–63.6]) than in the whole general adult population (12.7%; 95% CI: [9.2–16.2]), each of them being significantly (*P* < 0.05) different from the two others. Within the general adult population, the exclusion of workers in contact with ruminants resulted in a decreased seroprevalence rate (10.8%). Among all participants, only one seropositive veterinarian reported having had Q fever diagnosed by a physician in 2014.

## Discussion

The present study confirmed the possible spillover of *C. burnetii* from cattle to humans. In infected-cattle areas almost free from small-ruminants, the prevalence rate of antibodies-carrying subjects being higher than 10%, whatever the population considered. In addition, human infection with *C. burnetii* is primarily occupational: the estimated seroprevalence rates were much higher in cattle farmers and livestock veterinarians than in the general adult population.

In addition, these results suggest an exposure dose–effect relationship for the risk of seropositivity. The higher the number of sources and at-risk situations (e.g. abortions with high *C. burnetii* shedding), the higher the seroprevalence. Indeed, it is reasonable to assume an increasing gradient in terms of cumulative exposure between the general population, the cattle farmers group, and finally the veterinarians group. The lower prevalence in the general adult population, once the workers in contact with ruminants were excluded, supports this hypothesis. A similar ranking of seroprevalence rates among veterinarians and among cattle farmers was already reported in Denmark [8].

Overall, comparisons with other seroprevalence studies must be cautious, because of different contexts (e.g. outbreak *vs.* endemic situation; presence or absence of small ruminants in the study area); tests (e.g. IFA *vs.* enzyme-linked immunosorbent assay), cut-off used (e.g. low value associated with increased sensitivity, decreased specificity and larger observed seroprevalence) or lack of information (e.g. no mention that the general population may include people working in contact with ruminants). The dilution retained in the present study to consider a participant as positive was set at 1:16, whereas a higher cut-off value of 1:32 was used in some recent cross-sectional seroprevalence studies, especially those carried out in the Netherlands, e.g. [6]. However, these studies were conducted in a post-epidemic context, while ours was performed in an endemic situation where the infectious pressure was assumed to be much lower. Therefore, the cut-off value of 1:16 that we used was purposively chosen to be low – leading to an increased sensitivity of the IFA – to be able to detect infected persons with even very low IgG titres. The same choice was made by Chu *et al*. [11] to estimate seroprevalence among slaughterhouse workers in an endemic context in South Korea. Concerning the general adult population, the prevalence rate estimated here (12.7%) was in the range reported in studies carried out in Europe and North America (8–14%) [7]. Serological studies focusing on cattle farmers are scarce, and report either much lower estimates (<25%) [8, 12] or higher (>80%) [6, 13] than ours (56.3%). For livestock veterinarians, the prevalence rate found here (88.9%) was much higher than described in the literature to date (<65%) [8].

In addition, we cannot ensure the absence of selection bias having a possible impact on the estimates, while recruiting the participants in the three populations. Healthy blood donors are often considered to be poor representative of the general adult population for many chronic diseases. However, in the context of an airborne infection, we believe that this bias is highly reduced. In addition, these donors have been selected by quota sampling on age, gender and level of urbanity to make the sample as much as possible representative of the entire population of Finistère. Finally, as Q fever is often unknown of the public at large and mostly asymptomatic, it is very unlikely that the eligible donors might have chosen to participate because of a suspicion for themselves. For cattle farmers however, the risk of over-representation of those knowing at inclusion time that their dairy herd was positive towards *C. burnetii* infection cannot be excluded. This selection bias may have led to a possible over-estimation of the seroprevalence rate in this group. The livestock veterinarians were also volunteers, but, as all belonged to a highly exposed group, we assume the absence of selection bias in relation to Q fever.

The present study evidences that *C. burnetii* infection is endemic in humans in western France. This finding may be surprising. Indeed, as the study was carried out purposively in an area with very few notifications of acute Q fever cases (only one veterinarian declared having had Q fever in the past in our study), lower seroprevalence rates might have been expected. Several reasons could explain this apparent discrepancy. First, Q fever cases may have been underdiagnosed by physicians due to its nonspecific and mostly limited symptoms, especially in a non-epidemic context as in Western France. Second, Q fever cases, when diagnosed, may be underreported to the French reference laboratory, as Q fever is not a notifiable disease in France, even if the French Public Health authorities encourage physicians to send patient serum to the French reference laboratory for diagnostic confirmation. To support these assumptions, Q fever is reported to be diagnosed predominantly in the south-east of France, where the French reference laboratory is located: thus the physician's awareness towards Q fever is highly dependent on the presence of a reference laboratory nearby [3]. Third, clinical illness for *C. burnetii*, when acquired from infected cattle, has been also reported to be a rare event or with a mild course [8]. Studies report that the symptomatology found in *C. burnetii*-infected subjects may depend on the virulence of the involved strain [14] and also on the host adaptation: *C. burnetii* isolates originating from infected cattle would induce in human a more pronounced proinflammatory cytokine response compared to isolates from infected goats and sheep [15]. Fourth, the magnitude of the infectious pressure to which the subjects are exposed could be another variation factor. Indeed, the seasonal reproduction in small ruminants may lead to the exposure to larger sizes of inocula possibly inhaled, compared to cattle. Finally, it is still debatable whether or not the presence of antibodies in occupationally exposed people with frequent boosting is of clinical significance [16]. All these make compatible the co-existence of high seroprevalence and low incidence rates of Q fever cases among humans exposed to cattle.

The seroprevalence rates, estimated in this study conducted in an endemically infected area almost free from small ruminants, suggest that there is a specific risk for acquiring *C. burnetii* infection from cattle, mainly in occupational risk groups, but also for the general adult population. As already shown by Roest *et al*. in the Dutch context [17], comparison of strains could be performed in future research to definitively identify which types of animals (cattle *vs.* small ruminants *vs.* others) is responsible for *C*. *burnetii* infection in humans in such areas. Our finding strengthens the need to identify and quantify the impact of risk factors for *C. burnetii* infection in humans with the aim to determine specific preventive actions which could be advised to the human populations living in contact with or close to cattle.

## Data Availability

The data that support the findings of this study are available from the corresponding author.
